# Remobilization of *Sleeping Beauty *transposons in the germline of *Xenopus tropicalis*

**DOI:** 10.1186/1759-8753-2-15

**Published:** 2011-11-24

**Authors:** Donald A Yergeau, Clair M Kelley, Emin Kuliyev, Haiqing Zhu, Michelle R Johnson Hamlet, Amy K Sater, Dan E Wells, Paul E Mead

**Affiliations:** 1 Department of Pathology, St Jude Children's Research Hospital, 262 Danny Thomas Place, Memphis, TN 38105, USA; 2 Department of Biology and Biochemistry, University of Houston, Houston, TX 77204, USA

## Abstract

**Background:**

The *Sleeping Beauty *(*SB*) transposon system has been used for germline transgenesis of the diploid frog, *Xenopus tropicalis*. Injecting one-cell embryos with plasmid DNA harboring an *SB *transposon substrate together with mRNA encoding the *SB *transposase enzyme resulted in non-canonical integration of small-order concatemers of the transposon. Here, we demonstrate that *SB *transposons stably integrated into the frog genome are effective substrates for remobilization.

**Results:**

Transgenic frogs that express the *SB*10 transposase were bred with *SB *transposon-harboring animals to yield double-transgenic 'hopper' frogs. Remobilization events were observed in the progeny of the hopper frogs and were verified by Southern blot analysis and cloning of the novel integrations sites. Unlike the co-injection method used to generate founder lines, transgenic remobilization resulted in canonical transposition of the *SB *transposons. The remobilized *SB *transposons frequently integrated near the site of the donor locus; approximately 80% re-integrated with 3 Mb of the donor locus, a phenomenon known as 'local hopping'.

**Conclusions:**

In this study, we demonstrate that *SB *transposons integrated into the *X. tropicalis *genome are effective substrates for excision and re-integration, and that the remobilized transposons are transmitted through the germline. This is an important step in the development of large-scale transposon-mediated gene- and enhancer-trap strategies in this highly tractable developmental model system.

## Background

Amphibian model systems have provided a wealth of information on the molecular mechanisms controlling early vertebrate development. Frogs of the *Xenopus *genus are particularly well suited for embryological study as these animals adapt well to captivity and the females can be induced to lay large numbers of eggs throughout the year. The most commonly used amphibian model is the South African clawed frog, *X. laevis*. Genetic manipulation of this species is not practical due to the long generation time (> 1 year) and the pseudo-tetraploid nature of the genome. Another species of the *Xenopus *genus, *X. tropicalis*, shares the embryological advantages of its South African cousin and is better suited for genetic studies as it is a true diploid and has a relatively short generation time (approximately 6 months). The potential of applying modern genetics to this classical embryological model system has resulted in the rapid development of genomic tools for *X. tropicalis *in recent years (reviewed in [1,2]), and the publication of the genome sequence [3].

Our studies have focused on using the class II DNA 'cut-and-paste' transposable elements to modify the frog genome for gene- and enhancer-trapping and for insertional mutagenesis [4-9]. Transposable elements have been used for many years to experimentally modify the genomes of plants and invertebrates and, more recently, have been applied to vertebrate model systems [10,11]. Transgenesis with non-autonomous transposable elements offers advantages over other transgenic methodologies. First, transposable elements efficiently integrate into the target genomes. Second, as the transposon is excised from the donor plasmid prior to integration, plasmid sequences, which may cause epigenetic silencing [12,13], are not integrated at the targeted locus. Third, once integrated into the genome, the transposon transgene is an effective substrate for excision and re-integration (remobilization) following re-expression of the cognate transposase enzyme. The ability to remobilize transposons resident in the genome can be used for a variety of applications, including large-scale transposon 'hopping' screens using gene- or enhancer-trap constructs.

Remobilization of a non-autonomous transposon transgene is achieved by expressing the transposase enzyme in the same cell harboring the transposon. This can be achieved by simply injecting fertilized one-cell embryos from the outcross of transposon transgenic animals with mRNA encoding the transposase. As development proceeds, the injected mRNA is translated by the host cell and catalyzes the excision and re-integration reactions. This approach has been used successfully with the *Tol2 *transposon system in fish and frogs [7,14-16]. Another approach is to develop transgenic animals that express the transposase enzyme under the control of tissue specific promoters and to cross these animals with those that harbor a transposon substrate to generate double-transgenic progeny. This approach has been used very successfully for somatic remobilization of the *Sleeping Beauty *(SB) transposon to identify cancer genes in mice [17,18]. Outcross of the transposase enzyme and transposon substrate double transgenic animals can result in novel remobilization events in the progeny [19-23].

We, and others, have used a co-injection strategy with the *SB *[24] transposon system to generate transgenic *Xenopus *that express fluorescent proteins under the control of ubiquitous or tissue-specific promoters [4,6,25]. The integration events generated by this method in the frog are not caused by the simple transposition of the transposon from the plasmid into the frog genomic DNA. Analysis of the integration sites indicated that several copies of the transposon, and parts of the flanking plasmid sequence, are introduced at discrete loci as small-order concatemers. This unexpected non-canonical integration mechanism makes cloning the integration site complicated and time consuming [6]. Although the integration events generated by the co-injection strategy resulted in non-canonical integration, we next investigated whether *SB *transposons stably integrated into the *X. tropicalis *genome are effective substrates for remobilization. Using a double-transgenic strategy, we show that *SB *transposons in the frog genome can be remobilized following re-expression of the *SB *transposase and that the remobilized integration events occur via canonical transposition.

## Results

### Generation and analysis of transgenic *X. tropicalis *expressing *SB*10 transposase

**Figure 1:**
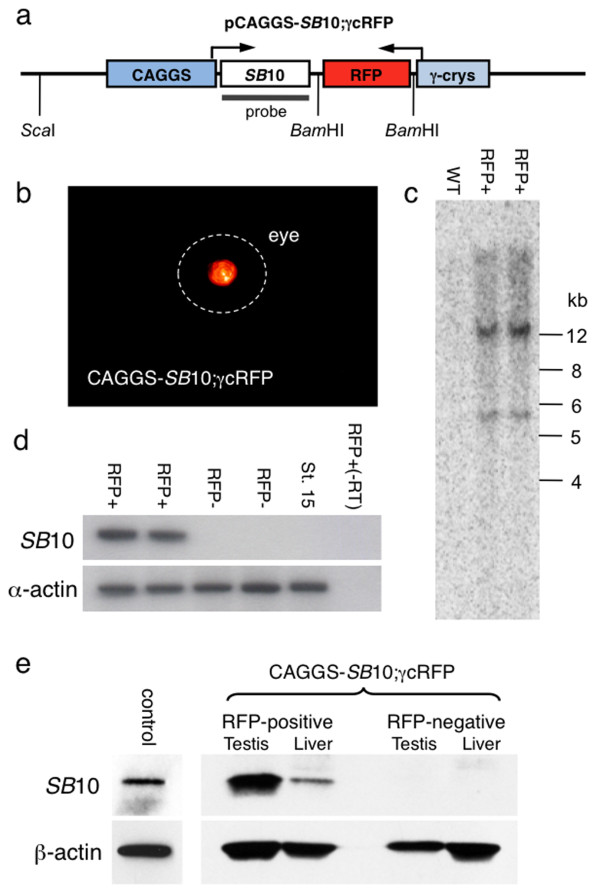
**Generation of a transgenic *Xenopus tropicalis *that expresses *SB*10 transposase**. **(a) **Schematic of the pCAGGS-*SB*10;γcRFP construct used to develop *SB *(*SB*10) transposase-expressing transgenic frogs. The two transgenes were cloned in a tail-to-tail orientation. Not to scale. **(b) **Red lens in the right eye of an adult F_1 _transgenic frog from outcross of founder CAGGS-*SB*10;γcRFP 2 M. The border of the eye is indicated by the dashed white line. **(c) **Southern blot analysis of genomic DNA harvested from RFP-positive and control animals indicated integration of multiple copies of the CAGGS-*SB*10;γcRFP linear transgene. The DNA was digested with *Bam*HI and the blot was probed with a radiolabelled *SB*10 cDNA probe (see schematic (a)). **(d) **RT-PCR analysis of *SB*10 expression in tadpoles. *SB *RNA was detected in RFP-positive tadpoles (+RFP) but not in RFP-negative (-RFP) progeny from CAGGS-*SB*10;γcRFP 2M. RNA from a wild-type tadpole was used as a negative control (St. 15). A mock reverse transcription reaction, without added RT, with RNA harvested from an RFP-positive tadpole (+RFP(-RT)) was used as a negative control. Primers for *X. tropicalis *α-actin were used as a control for RNA recovery. **(e) **Western blot analysis of *SB *transposase expression in tissues harvested from adult transgenic frogs. A monoclonal antibody to *SB *was used to demonstrate abundant transposase expression in the testis and liver of RFP-positive adults, but not in the RFP-negative siblings. Protein lysates prepared from tadpoles injected with *SB*10 mRNA at the one-cell stage were prepared at stage 15 (control lane). The blots were stripped and re-probed with a monoclonal antibody that recognizes *Xenopus *α-actin. PCR: polymerase chain reaction; RFP: red fluorescent protein; RT: reverse transcriptase; SB: *Sleeping Beauty*.

A transgenic *X. tropicalis *line was engineered to express the *SB*10 transposase under the control of a synthetic regulatory element, chicken β-actin promoter coupled with a cytomegalovirus enhancer (CAGGS [26]) [27]. To track the inheritance of the *SB*10 transgene, a *X. laevis *γ1 crystallin-red fluorescent protein (RFP) [28] reporter was cloned downstream of the CAGGS-*SB*10 transgene in a head-to-head orientation (Figure 1a). The presence of the linked γ1 crystallin-RFP reporter allows screening for the CAGGS-*SB*10 transgene based on the presence of red eyes (Figure 1b). We used the simple linear plasmid DNA injection method described by Etkin and Pearman to generate the transgenic *SB *transposase-expressing frogs [29]. Injected embryos were scored for the presence of RFP expression in the lens, and RFP-positive tadpoles (27 RFP-positive from 570 injected, 4.7%) were raised to adulthood. A single founder (CAGGS-*SB*10;γcRFP 2 M), from a total of five animals outcrossed to date, was identified. Outcross of male founder CAGGS-*SB*10;γcRFP 2M with a wild-type female resulted in 779 RFP-positive tadpoles from a total of 3,333 offspring (23.4%). The non-Mendelian inheritance of the transgene indicates that the germline of the CAGGS-*SB*10;γcRFP 2M founder was mosaic for the transgene. Subsequent outcross of F_1 _animals derived from CAGGS-*SB*10;γcRFP 2M resulted in the expected 50% of the progeny expressing the dominant lens-specific RFP reporter (in a representative F_1 _outcross there were 239 RFP-positive tadpoles from a total of 479, 49.9%). Southern blot analysis of RFP-positive tadpoles indicated that several copies of the transgene were integrated at a single locus in the founder (Figure 1c). Reverse transcriptase (RT)-PCR and Western blot analyses were used to verify that *SB*10 transposase was expressed in the transgenic line. RT-PCR analysis showed that RFP-positive tadpoles at stage 40 [30] express mRNA encoding the *SB *transposase enzyme (Figure 1d). As expected, sibling tadpoles that did not express the RFP reporter in the lens were also negative for *SB*10 mRNA expression. In adults, robust expression of *SB *transposase was detected in protein lysates prepared from testes harvested from RFP-positive male frogs, but not from RFP-negative animals (Figure 1e). *SB*10 is also expressed in the liver of the transgenic frogs, but not in the RFP-negative littermates.

### Generation of double-transgenic 'hopper' frogs

**Figure 2:**
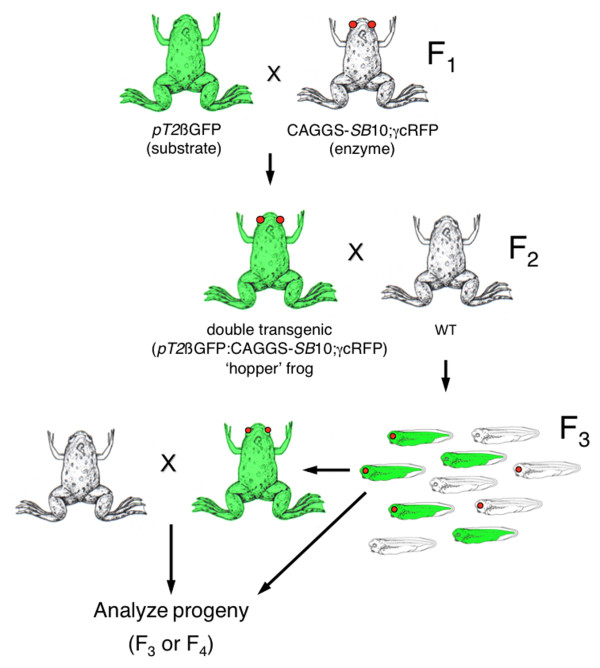
**Breeding strategy to generate double-transgenic hopper frogs**. The F_2 _hopper frogs were outcrossed with wild-type animals and the progeny was scored for GFP and RFP expression. The GFP-positive/RFP-negative F_3 _progeny were either raised to adulthood for outcross or genomic DNA was harvested after stage 45 for molecular analyses. GFP: green fluorescent protein; RFP: red fluorescent protein.

The CAGGS-*SB*10;γcRFP 2M line was outcrossed with *SB *transposon transgenic animals that express GFP under the control of the CAGGS promoter (*pT2*βGFP [6]). Double-transgenic F_2 _'hopper' frogs (ubiquitous GFP and lens-specific RFP) were outcrossed with wild-type frogs and the progeny (F_3_) were either analyzed for remobilization events or raised and outcrossed (Figure 2). Five independent substrate donor lines were used to generate double-transgenic hopper lines for this study. As the methodology for the generation and analysis of the hopper lines is the same for each donor locus, two donor lines (*pT2*βGFP 8 F and 7 M) will be described in detail below.

### 8F hoppers

The *pT2*βGFP 8F founder harbors two independently-segregating alleles: a concatemer of three *SB *transposons integrated at a single locus on scaffold 57, at base number 2456981 (**57**:2456981) of the JGI *X. tropicalis *genomic sequence v4.1 assembly, and another allele with a single-copy transposon integration [6]. Thus, the F_2 _hopper frogs inherited either one, or both, of the 8F integration events. Southern blot analysis of progeny from 8Fhopper♂35 indicated that this double-transgenic hopper had inherited the trimeric concatemer of *pT2*βGFP on scaffold 57 alone. Double-transgenic (RFP+/GFP+) progeny (F_3_) from the outcross of 8Fhopper♂35 were raised and outcrossed, and the resulting progeny (F_4_) were analyzed for modification of the parental *pT2*βGFP locus (Figure 2).

**Figure 3:**
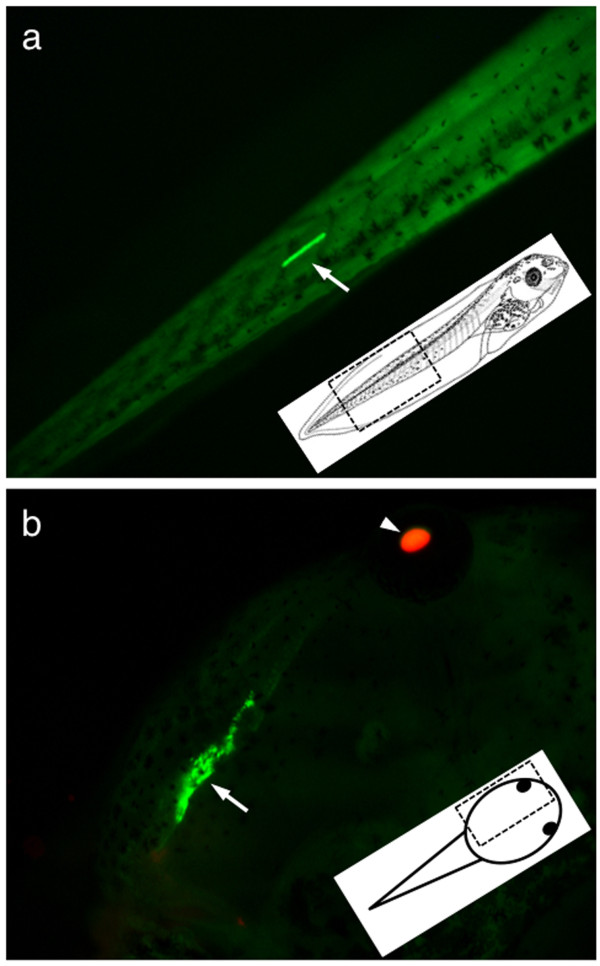
**Somatic remobilization of *pT2*βGFP in double transgenic tadpoles**. Outcross of double transgenic hopper frogs resulted in progeny that inherited both transgenes. In rare instances, we identified double transgenic tadpoles that express intense levels of the GFP transgene reporter in individual cells or in small groups of cells. The change in GFP expression seen in these somatic cells is likely to be due to sporadic remobilization of the *pT2*βGFP transposon and the change in GFP intensity is likely due to the influence of the local chromatin environment at the novel integration site. The region of each tadpole shown (dashed box) is indicated on the cartoon inset. **(a) **Tail of a double transgenic tadpole with a single muscle cell expression intense GFP (arrow). **(b) **Double transgenic tadpole with high-level GFP expression in a subset of cells in the brachial cartilage (arrow). The immobilized tadpole was also photographed using a dsRED filter and the two images were overlaid to demonstrate that this animal had inherited the CAGGS-*SB*10;γcRFP transgene (RFP expression in the lens is indicated by the white arrowhead). GFP: green fluorescent protein; RFP: red fluorescent protein.

**Figure 4:**
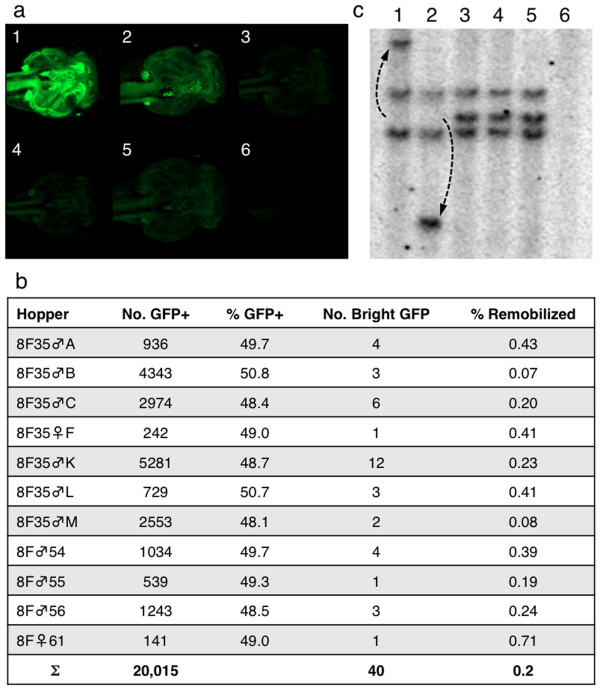
**Excision and re-integration of *SB *transposons in the progeny of double-transgenic hopper frogs**. **(a) **GFP expression in sibling tadpoles derived from the outcross of an 8F hopper frog. Tadpoles 1 and 2 are significantly brighter than their GFP-positive siblings (tadpoles 3, 4 and 5). Tadpole 6 is a GFP-negative tadpole. Dorsal view, with anterior facing towards the right. **(b) **Representative data for the outcross population of 8F hopper frogs. Table includes data from breeding four F2 (8F♂54, 8F♂55, 8F♂56 and 8F♀61) and seven F3 (8F35♂A, B, C etc.) double-transgenic hoppers with wild-type frogs. The outcross progeny were scored for GFP expression and the GFP-bright progeny were either harvested for integration site analysis or raised to adulthood and outcrossed. A range of apparent remobilization activity from 0% to 0.7% was observed in individual 8F hopper frogs, with an average rate of two remobilization events per thousand GFP-positive progeny (0.2%). **(c) **Southern blot analysis of genomic DNA harvested from the progeny of double transgenic 8F hopper frogs. Genomic DNA was digested with *Bgl*II and the blot was probed with a radiolabelled GFP cDNA probe. DNA harvested from tadpoles in lanes 3, 4 and 5 have the same banding pattern as the parental *pT2*βGFP 8F founder line. Lanes 1 and 2 show example of remobilization of an *SB *transposon. The dashed arrow indicates the change in the mobility of the transposon-harboring *Bgl*II fragment. Lane 6 contains DNA from GFP-negative siblings. GFP: green fluorescent protein; *SB*: *Sleeping Beauty*.

Observation of the GFP expression in the hopper outcross populations indicated that, in most cases, the GFP expression of the progeny was identical to that of the 8F founder, suggesting that the parental *SB *transposon locus was intact. In a small number of the outcross progeny, we observed markedly different GFP expression in either small populations of cells within the tadpole (Figure 3a, b) or in whole tadpoles (Figure 4a, b; 40 from 20,015 GFP-positive tadpoles). We reasoned that the change in GFP expression might result from the modification of the parental *pT2*βGFP locus in the remobilized progeny. Embryos with small subsets of cells with increased GFP intensity likely represent stochastic transposase activity in somatic tissues (somatic remobilization (Figure 3a, b)). An organism-wide change in GFP intensity (Figure 4a) likely represents modification of the parental transposon donor locus during gametogenesis that is passed on to the resulting progeny. Remobilization of a transposon from the donor locus to a novel site will likely alter the local epigenetic environment of the transgene, and also subject the re-integrated transposon to the influence of nearby gene regulatory sequences that differ from the parental locus.

Genomic DNA harvested from GFP-positive progeny from double transgenic (*pT2*βGFP8F:CAGGS-*SB*10;γcRFP) 8F hopper frogs was analyzed by Southern blot. Digestion of genomic DNA from *pT2*βGFP 8F tadpoles with *Bgl*II resulted in three bands when the blot was hybridized with a GFP probe (Figure 4c). Changes in the Southern blot hybridization pattern were used to determine whether the parental concatemer had been altered by expression of the *SB *transposase. Analysis of progeny from the outcross of F_3 _double transgenic hopper frogs with wild-type animals indicated that most of the progeny had inherited the unaltered *pT2*βGFP 8F parental concatemer (Figure 4c; lanes 3, 4 and 5). Examples of germline remobilization of the *pT2*βGFP transposon from 'GFP-bright' tadpoles (Figure 4a; tadpoles 1 and 2) harvested from the outcross of 8Fhopper♂58 are shown in Figure 4c (lanes 1 and 2, dashed arrows). This data indicates that, as predicted, the GFP-bright individuals in the outcross population of the hopper frogs represent tadpoles that have modified the parental transposon donor locus. Thus, remobilized animals can be identified in the outcross population by simply observing the tadpoles for changes in GFP intensity. Outcross of eleven 8F hopper double transgenic frogs indicated that the frequency of remobilized progeny varied from 0.07% to 0.71% (Figure 4b). The variation in the remobilization activity between individual hopper frogs likely reflects subtle differences in epigenetic modification of the substrate and enzyme transgenes in each animal that may alter the activity of the excision and reintegration reactions.

**Figure 5:**
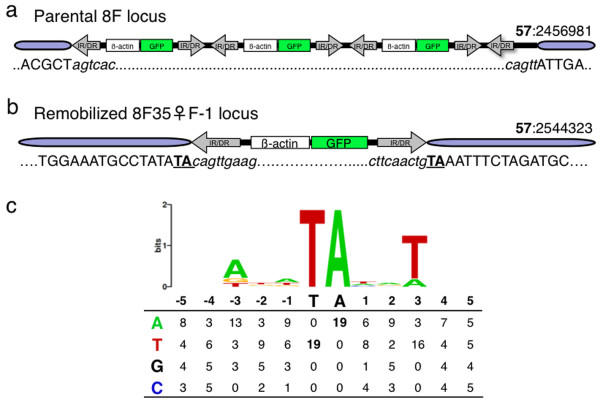
**Integration site analysis of remobilized *SB *transposons**. **(a) **Schematic representation of the 8F donor locus showing the predicted orientation of the trimeric concatemer in scaffold 57. This injection-mediated integration event occurred by a non-canonical mechanism. (Not to scale.) **(b) **Schematic representation of the novel integration event in the remobilized tadpole shown in Figure 4b. EPTS LM-PCR was used to clone the sequence flanking the 5' end of the *pT2*βGFP transposon and the 5' and 3' flanking sequences were verified using PCR primers designed to the scaffold sequence. The novel integration event occurred on the same scaffold (57 at position 2544323 bp) of the Joint Genome Institute *X. tropicalis *genome sequence assembly v4.1 as the 8F transposon donor locus (**57**:2456981) and represented a local hop. The sequence of the *SB *transposon and genomic DNA junctions (arrows) indicated that the remobilization event occurred via a canonical transposition reaction. The *pT2*βGFP transposon is flanked by the expected TA dinucleotide target site duplication (TSD; bold underlined), and the transposon is inserted precisely, without any flanking plasmid sequence from the donor site. The genomic DNA sequence of scaffold 57 is capitalized and the transposon sequence is in lowercase italics. (Not to scale.) **(c) **The preferred sequence for *SB *transposon re-integration in the *X. tropicalis *genome. Weblogo analysis http://weblogo.berkeley.edu for the five base pair sequence flanking the TA target site. The relative size of the letters indicates the strength of the information on the y-axis, with the maximum indicated by two bits. The table shows the base distribution of the *pT2*βGFP transposon re-integration target sites. EPTS LM-PCR: extension primer tag selection linker-mediated polymerase chain reaction; PCR: polymerase chain reaction; *SB*: *Sleeping Beauty*.

**Figure 6:**
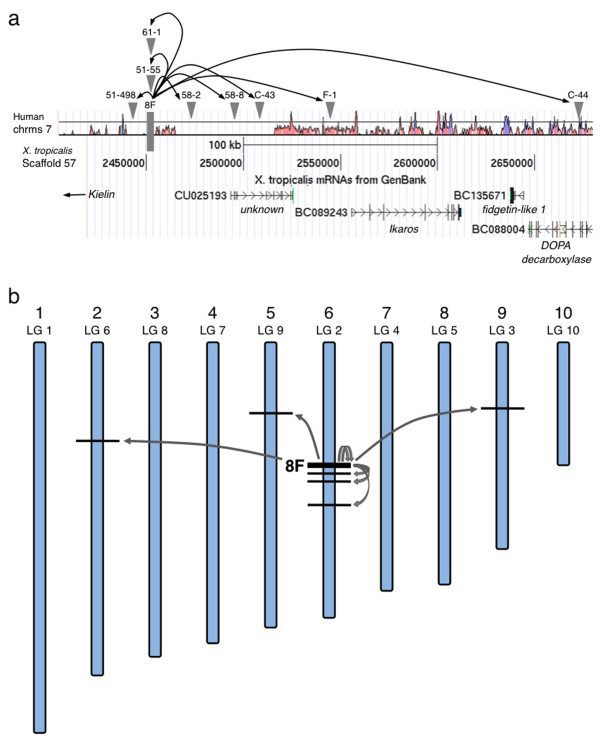
**Schematic representation of remobilized *SB *transposons in the *X. tropicalis *genome**. **(a) **Local hopping on *X. tropicalis *chromosome 6 depicts the integration sites for eight local (< 200 kb) remobilization events (hops). (Not to scale.) This region of *X. tropicalis *chromosome 6 is syntenic with human chromosome 7. The Vista http://genome.lbl.gov/vista alignment shows regions of homology between the frog and human genomic sequences; pink represents non-coding regions and blue represents conserved exons. The position of the 8F donor concatemer is indicated by the grey box. The remobilized transposition events are depicted by the grey triangles. The position and orientation of predicted genes near the 8F locus are depicted in the lower section of the panel. **(b) **Schematic representation of the *X. tropicalis *chromosomes indicating the distribution of remobilized *SB *transposons. The parental 8F donor site is on chromosome 6 (thick line). The predicted loci of the remobilized transposons are depicted by the thin black lines. Approximately 80% of the remobilization events occur near the donor locus (local hopping), and the remaining 20% are distributed randomly throughout the genome. *SB*: *Sleeping Beauty*.

Analysis of the cloned flanking sequences of the parental locus (**57**:2456981) from the remobilized tadpoles (Figure 4c; lane 1 and 2) showed no sequence change, indicating that the remobilized transposon was excised from within the donor concatemer (data not shown). Extension primer tag selection linker mediated-PCR (EPTS LM-PCR) and standard genomic PCR [6,8] were used to clone the integration sites of the novel bands. The re-integration event from tadpole 8Fhopper♂58-1 had occurred on the same scaffold as the parental integration site, and thus represented a 'local hop' (Table 1 and Figure 5; tadpole 8Fhopper♂58-1). Genomic PCR and sequencing was used to verify both the 5'- and 3'-ends of the novel insertion site. The integration site of the remobilized *pT2*βGFP transposon is at **57**:2491386 and is 34,405 bp away from the parental locus on chromosome 6 (Figure 5b). Sequence analysis of the integration site indicated that the remobilization event was catalyzed by a canonical transposition event. That is, the transposon inserted precisely at the predicted boundary of the indirect repeat/direct repeats (IR/DRs). Furthermore, the integrated transposon is flanked by the expected TA dinucleotide target site duplication catalyzed by *SB *transposase [31-33]. Thus, unlike the co-injection method used to generate the *pT2*βGFP founder lines that results in unexpected concatemer formation (Figure 5a, [6]), the remobilization events catalyzed by re-expression of *SB *transposase are via canonical transposition (Figure 5b). To date, we have identified 40 remobilization events, based on differences in GFP expression intensity, from 20,015 GFP-positive tadpoles from the outcross of 8F hopper frogs (Figure 4b). Southern analysis has confirmed excision and re-integration of a *SB *transposon from the parental locus and yields an apparent remobilization frequency of approximately 0.2%.

**Table 1:** Integration site analysis for remobilized progeny from 8F hoppers.

**Tadpole**	**Right indirect repeat/direct repeats flanking sequence**	**Integration site**	**5' flanking gene**	**3' flanking gene**
Parental 8F	GCAACGCTagtcac...cagttATTGATT^a^	**57**:2456981	*pSST739*	*C7orf72-like*
	
8F♂51-498	GCGCAATACTATTA**TA***cagttgaagtcgg*	**57**:2452131	*pSST739*	*C7orf72-like*
8F♂51-55	CGGGCCATGATGTA**TA***cagttgaagtcgg*	pT2βGFP^b^	*pSST739*	*C7orf72-like*
8F♀61-1	GTGGAGCTCTGAAA**TA***cagttgaagtcgg*	pT2βGFP^b^	*pSST739*	*C7orf72-like*
8F♂58-2	AAAGGCAACACGCG**TA***cagttgaagtcgg*	**57**:2470830	*pSST739*	*C7orf72-like*
8F♂58-8	GAATCTCTGTGATC**TA***cagttgaagtcgg*	**57**:2491386	*pSST739*	*C7orf72-like*
8F♂C-43	CAGAGCTAGATATA**TA***cagttgaagtcgg*	**57**:2507804	*C7orf72-like*	*C7orf72-like*
8F35♀F-1	TGGAAATGCCTATA**TA***cagttgaagtcgg*	**57**:2544323	*C7orf72-like*	*Ikaros*
8F35♂C-44	AAGAAAGCACTTGG**TA***cagttgaagtcgg*	**57**:2672369	*Dopa decarboxylase*	*Dopa decarboxylase*
8F♀60-1	CCCCCTTCGGTGAT**TA***cagttgaagtcgg*	**57**:2897938	*Grb10*	*Cordon-bleu*
8F35♂A-207	ACAAACGGGCCATG**TA***cagttgaagtcgg*	**57**:2991209	*Grb10*	*Cordon-bleu*
8F♂58-9	TATCTAAACAAAGT**TA***cagttgaagtcgg*	**588**:587502	*Cam-PDE 1C*	*Cam-PDE 1C*
6265	TCACTACATATTTC**TA***cagttgaagtcgg*	**294:**394261	*PTPRM*	*PTPRM*
8F♂58-3	TAAGAATTAATAGT**TA***cagttgaagtcgg*	**250**:752306	*c-Fyn*	*c-Fyn*
8F35♂C-35	TATAAATAAAGATA**TA***cagttgaagtcgg*	**223**:803513	*Connexin 31.1*	*C1orf94-like*
8F♂B-203	AAGGCAGTCAGTTA**TA***cagttgaagtcgg*	**15**:2263789	*RDC-1*	*COP9*
8F35♂A-205	GATAACTCTTAAGT**TA***cagttgaagtcgg*	**484**:822241	*Amphiphysin*	*Amphiphysin*

Sequences flanking the novel transposon insertion site were cloned using extension primer tag selection linker mediated-PCR. The remobilization events are mediated by canonical transposition reactions and the transposon sequence (*italics*) is flanked by the characteristic TA dinucleotide target site duplication (bold). The flanking sequence (depicted in capital letters) was used to interrogate the *X. tropicalis *genome sequence database (Joint Genome Institute *X. tropicalis *genome sequence assembly v4.1; [3]) to assign the integration sites to the genomic sequence scaffolds. The genes flanking the novel integration site are listed.

^a^The parental integration site on scaffold 57 was mediated by a non-canonical mechanism. ^b^Two remobilization events showed reintegration of the excised transposon into the donor concatamer locus on scaffold 57.

Pre-sorting tadpoles based on GFP intensity may underestimate the total remobilization activity if the re-integration event resulted in GFP expression that was not markedly different from the parental expression. To test this, we outcrossed a double transgenic hopper frog (8Fhopper♂51) and analyzed all of the GFP-positive progeny by Southern blot. The 8Fhopper♂51 frog inherited both of the *pT2*βGFP transposon alleles from the 8F founder. The progeny from this 8Fhopper♂51 outcross displayed GFP expression patterns and intensities that were indistinguishable from that of the parental alleles (data not shown). From the 677 GFP-positive progeny analyzed by Southern blot, we identified four excision-only events and two remobilizations. Samples of genomic DNA where changes were evident by Southern blot analysis were used in EPTS LM-PCR to clone the integration site of the remobilization events. In this experiment, the remobilization frequency was 0.3% (two remobilization events out of 677 GFP-positive tadpoles). These data indicated that the actual remobilization frequency may be somewhat higher than that estimated by simple visual inspection of the GFP-positive progeny. The observed rate of excision-only events in this outcross population was 4 out of 677, that is, 0.6%.

Scoring the outcross progeny of hopper frogs for changes in GFP intensity may also overestimate the remobilization frequency, as this method may not distinguish between remobilization events and excision-only events. We analyzed 25 GFP-bright tadpoles from the outcross of 8F and 7M (see below) hopper frogs, by Southern blot analysis and by cloning the novel insertion sites by EPTS LM-PCR. Only one GFP-bright tadpole had an excision-only modification of the parental transposon donor locus (4%); 24 GFP-bright tadpoles (96%) had re-integration events that were evident by novel bands on the Southern blot and by cloning the sequences flanking the canonical re-transposition events. Thus, while it is possible to identify excision-only events by changes in GFP expression, the vast majority of GFP-bright progeny represent re-integration events.

Remobilization of transposons resident in the genome may result in chromosomal rearrangements near the donor locus [34-41]. In mice, germline remobilization of *SB *transposons from a high-copy number (approximately 30 copies) concatemer resulted in frequent alteration of the genomic sequences flanking the transposon donor locus; nine out of nine remobilized pedigrees examined displayed genomic alterations spanning 10^5 ^bp to 10^7 ^bp flanking the donor site [41]. To determine whether *SB *remobilization in the frog resulted in similar genomic alterations near the donor locus, we examined the sequences flanking the 8F donor locus by PCR. Genomic DNA samples from eight remobilization events and eight excision-only events were used to amplify the sequences flanking the 5' and 3' ends of the 8F concatemer on chromosome 6. In each case, genomic PCR using primers that amplify the 5' and 3' junctions of the 8F locus generated the appropriate sized products (data not shown), indicating that the sequences directly flanking the donor locus are intact following excision of *pT2*βGFP transposons from the donor site.

Sequence analysis of the re-integration target sites indicated a similar base distribution flanking the canonical TA dinucleotide to that observed with *SB *integration in mammalian genomes [42,43] (Figure 5d). Transposons of the *Tc1/mariner *family, including *SB*, integrate at TA dinucleotides. The consensus sequence for *SB *integration in frogs, as in mammals, is a palindromic ATA**TA**TAT sequence, where the canonical TA target is in bold, although none of the re-integration events observed in the frog have this exact palindrome.

Cloning the integration sites of the novel loci indicated that the remobilized transposons frequently integrate near the parental locus (Figures 5 and 6; 12 out of 15 classed as local hopping, 80%). In two cases, we identified remobilization events that had re-integrated within the parental transposon concatemer on scaffold 57 (Table 1 and Figure 6a). The scaffold identity was used to 'map' the chromosomal location [44] of the novel integration events and showed that, while local hopping was more frequent, re-integration on other chromosomes was also detected (Figure 6b; three out of fifteen (20%) of integrations are on different chromosomes).

GFP-bright progeny from the outcross of 8F hopper frogs were raised to the adult stage, and outcrossed to demonstrate that the remobilized transposon alleles are stably transmitted through the germline. Genomic DNA was harvested from GFP-positive and GFP-negative siblings and used for Southern blot analysis and for cloning the novel integration site by EPTS LM-PCR. For example, remobilized female frog 62E3 produced GFP-bright progeny and integration site analysis showed a single copy of the *pT2*βGFP transposon on scaffold 140 (**140**:1237072). The novel re-integration event was on the same chromosome as the donor locus (chromosome 6, linkage group 2), approximately 1 cM from the parental 8F concatemer, and represents a local hop (data not shown). The 62E3 integration event was in the 3' UTR of a muscle-related coiled coil protein (GenBank accession number XM_002935280.1) gene.

### 7M hoppers

**Figure 7:**
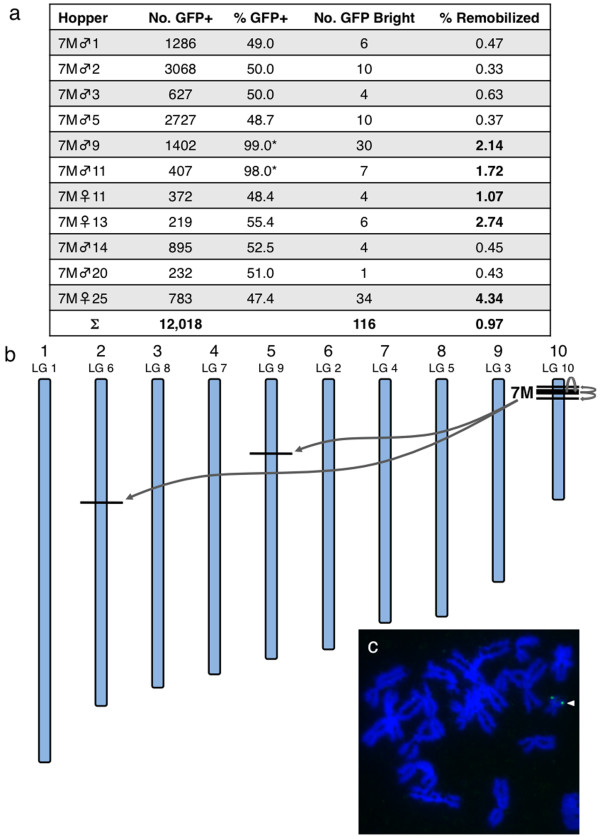
**Transposon hopping from the 7M donor locus**. **(a) **The outcross progeny from nine 7M hopper adults were scored for changes in GFP intensity indicative of transposon remobilization. The 7M donor locus contains a concatemer of approximately eight to ten *pT2*βGFP transposons on chromosome 10. The remobilization frequency is expressed as a percentage of GFP bright tadpoles observed in the GFP-positive outcross population. 7M hopper frogs 7M♂9 and 7M♂11 are 'functionally homozygous' for both the transposon substrate allele and the transposase enzyme transgene (see text for details), and display higher remobilization activity (approximately 1.8%) than their 'heterozygous' male hopper littermates (7M♂1, 7M♂2, 7M♂3, 7M♂5, 7M♂14, 7M♂20; approximately 0.44%). Outcross of 7M hopper 7M♀25 produced a remobilization rate of > 4%. **(b) **Schematic representation of the *X. tropicalis *chromosomes indicating the distribution of remobilized *SB *transposons. The parental 7M donor site (thick line) is located on scaffold 38 which maps to chromosome 10. Remobilization of discrete transposons away from the 7M donor locus is represented by the grey arrows. (Not to scale.) **(c) **Fluorescence *in situ *hybridization of metaphase chromosomes verifies that the 7M parental donor locus is located near a telomere of *X. tropicalis *chromosome 10. GFP: green fluorescence protein; *SB*: *Sleeping Beauty*.

The *pT2*βGFP 7M founder had a concatemer of 8 to 10 *pT2*βGFP transposons at a single locus within a repeat on scaffold 38 (Linkage Group 10, chromosome 10), and mapped, by fluorescence *in situ *hybridization (FISH) analysis, near a telomere on chromosome 10 (Figure 7). Double transgenic 7M hopper frogs were generated by breeding heterozygous *pT2*βGFP 7M F_1 _frogs with heterozygous CAGGS-*SB*10;γcRFP 2M F_1 _frogs, and the progeny were sorted for GFP-positive and RFP-positive expression. The double-heterozygous 7M hopper frogs were outcrossed with wild-type animals and remobilization events were scored in the progeny by observing the outcross population for changes in GFP expression. To date, ten 7M hoppers have been outcrossed and 112 remobilized (GFP-bright) tadpoles have been identified from 11,646 GFP-positive progeny (Figure 7a). Genomic DNA from several GFP-bright tadpoles was analyzed by Southern blot, and this data verified that the banding pattern had changed from the parental 7M pattern, indicative of transposon remobilization. The novel integration sites were cloned (Table 2), and sequence analysis confirmed that the remobilized transposons had re-integrated via canonical *SB*-mediated transposition (data not shown). The average apparent rate of remobilization was approximately 1%, and is five-times higher than that observed for the 8F hopper animals. The higher rate of remobilization observed in the 7M hoppers compared to the 8F hoppers may be due to the increased number of potential substrate transposons in the donor concatemer (three for 8F compared with 8 to 10 for 7M). A range of remobilization activities, from 0% to 5%, was noted between the different 7M hopper frogs. The 7M hoppers were produced by breeding frogs that were heterozygous for the *SB*10 enzyme transgene with frogs that were heterozygous for the *pT2*βGFP 7M allele. Double-heterozygous males (7Mhopper♂1, 7Mhopper♂2, 7Mhopper♂3, 7Mhopper♂5, 7Mhopper♂14, 7Mhopper♂20) produced offspring with an average remobilization frequency of approximately 0.44%. The frequency of GFP-positive progeny in the outcrosses from these males was approximately 50%, as expected for the Mendelian inheritance of a heterozygous dominant allele. Two 7M hopper male frogs (7Mhopper♂9 and 7Mhopper♂11) produced a much higher rate of remobilized (GFP-bright) tadpoles than their siblings (approximately 1.9% compared with 0.44% for male sibling hoppers). Intriguingly, these animals appear to be homozygous for both the enzyme (CAGGS-*SB*10;γcRFP) and substrate (*pT2*βGFP) transgenes; 100% of the outcross progeny were RFP-positive and nearly all (> 98%) were also GFP-positive. Southern blot analysis of the outcross progeny indicated that all of the GFP-positive animals (n = 107 for 7Mhopper♂9; n = 114 for 7Mhopper♂11) had inherited the 7M concatemer, and the banding pattern was identical to the parental locus. The GFP-bright tadpoles in the outcross populations of 7Mhopper♂9 and 7Mhopper♂11 showed changes in parental 7M locus indicative of excision and re-integration of a transposon from the substrate donor locus. The rare (< 2%) GFP-negative tadpoles observed in the outcross populations did not inherit the *pT2*βGFP transgene as determined by Southern blot and genomic PCR for GFP sequences (data not shown). The unexpected non-Mendelian inheritance of hoppers 7M♂9 and 7M♂11 is unexplained; however, this data suggests that increasing the copy number of the transposon substrates in the hopper lines may increase the remobilization frequency observed in the outcross population.

**Table 2:** Integration site analysis for remobilized progeny from 7M Hoppers

Tadpole	Right IR/DR flanking sequence	Integration site	5' flanking gene	3' flanking gene
Parental 7M	TGTTAGTTATTACTTA*cagttgaagtcgg*	**38**:3796832	*EDEM2*	*PHF20*

E208-91	TCATTATCAGTATA**TA***cagttgaagtcgg*	**38:**552620	*EMILIN3*	*EMILIN3*
3-1	GCTACTCACACAGT**TA***cagttgaagtcgg*	**13:**899336	*SPRY2*	*NDFIP2*
2-7M	TGTTACTGGGCACT**TA***cagttgaagtcgg*	**3:**3883685	*MYNN*	*MDS1*
A21A-1	AGTGTGTAGCTATA**TA***cagttgaagtcgg*	**909**:49452	*GBP3*	*GBP3*
C2AB-5	CGGGCCATGATGTA**TA***cagttgaagtcgg*	Repeat	-	-
E2F3-95	GCACATAATACACA**TA***cagttgaagtcgg*	Unknown	-	-
E220-248	CATG**TA***cagttgaagtcgg*	Unknown	-	-

Sequences flanking seven novel transposon insertion sites from 7M hoppers were cloned using extension primer tag selection linker mediated-PCR. The transposon sequence (*italics*) is flanked by the characteristic TA dinucleotide target site duplication (bold). The flanking sequence (depicted in capital letters) was used to interrogate the *X. tropicalis *genome sequence database (Joint Genome Institute *X. tropicalis *genome sequence assembly v4.1; [3]) to assign the integration sites to the genomic sequence scaffolds. The genes flanking the novel integration site are indicated.

To date, four 7M female hopper frogs have been outcrossed, and the mean remobilization rate from these animals is 2.54%. This may reflect individual differences in excision and reintegration activities between different hopper animals, or it may indicate that remobilization, driven by the CAGGS-*SB*10 transgene, is more efficient in the female germline (mean 2.54%; n = 4) than in the male germline (mean 0.76%; n = 9, unpaired Student's *t*-test, *P *= 0.0088, degrees of freedom, 11). With the 8F hoppers, we observed a modest increase in the mean remobilization efficiency with the female hoppers (0.56%; n = 2) compared to the male hoppers (0.25%; n = 7); however, due to the small sample size, this may not be statistically significant (Student's *t*-test *P *= 0.25, degrees of freedom, 1).

The 7M *pT2*βGFP concatemer is located on scaffold 38 that maps to chromosome 10 (Figure 7b and 7c). Genomic DNA harvested from representative GFP-bright tadpoles from the 7M hopper outcrosses was analyzed by Southern blot and the novel integration sites were cloned by EPTS LM-PCR. As noted for the remobilized 8F hopper progeny above, the novel integration events from the 7M hoppers were canonical *SB*-mediated transposition events. As determined for the remobilization events from the 8F hopper frogs, a strong bias for local re-integration was observed for the 7M hoppers; re-integration events on the same scaffold (scaffold 38) as the transposon donor were cloned from the GFP-bright tadpoles.

## Discussion

### *SB *transposons can be remobilized in *X. tropicalis*

Here, we demonstrate that *SB *transposons integrated into the frog genome are effective substrates for remobilization following re-expression of the *SB *transposase. Unlike the integration events observed in the co-injection strategy that were mediated by a complex non-canonical mechanism, the remobilized *SB *transposons re-integrated via canonical *SB*-mediated transposition. The observed frequency of excision and subsequent re-integration of the parental *pT2*βGFP transposon was low (on average, less than 1%). Our data in *X. tropicalis *is similar to that observed in other *in vitro *[45] and *in vivo *[19,46,47] systems, where low-copy number transposon donor sites served poorly as substrates for remobilization. In mammals, increasing the number of transposon substrates by using donor sites that contain high-order concatemers resulted in increased remobilization activity [19,46]. For example, in AB1 embryonic stem cells, a single *SB *transposon was 'knocked in' to the *Hprt *gene on the mouse X chromosome and subsequent transient expression of *SB *transposase (*SB*10) resulted in a transposition rate of *circa *3.5 × 10^-5 ^events per cell per generation [45]. In mice, low-copy number *SB *transposon donor sites result in a low frequency of remobilization events that are passed through the germline [19,47]. For example, single-copy *SB *transposon donors result in novel re-integration sites in approximately one embryo in every one hundred (around 1%) in an outcross of double transgenic 'seed' mice [47]. Increasing the number of transposon substrates by using donor sites that contain high-order concatemers resulted in increased remobilization activity; however, Geurts and colleagues noted that there was not a linear correlation between donor site copy number and remobilization activity [47]. This suggests that other factors, such as the methylation status, and other local chromatin-environment factors, may also influence the ability of integrated *SB *transposons to serve as substrates for remobilization. Horie and colleagues observed that low-copy number *SB *transposon concatemers served very poorly as substrates for remobilization in mice, and that the presence of more copies of the *SB *transposon in the concatemer acted synergistically to increase the frequency of excision and re-integration. With a donor site that contained around 20 copies of the transposon substrate, a remobilization rate of 1.25 transpositions per genome per animal (125%) was observed [19]. Keng and colleagues also reported a similar transposition frequency when using double transgenic mice that contained either 20 copies (1.16 transpositions per GFP-positive mouse) or 100 copies (1.14 transpositions per GFP-positive mouse) of the substrate transposon in the donor concatemer [46].

In this study, we used low-copy number donor sites as substrates for remobilization as the integration events observed with the plasmid-mRNA co-injection strategy were mediated by a complex, non-canonical integration mechanism [6]. We reasoned that, if a similar non-canonical mechanism were used in the remobilization step, starting with a simple substrate would help facilitate cloning of the remobilization event. Although the remobilization frequency observed in the outcross of the double transgenic frogs is low, the re-integration events are canonical *SB*-mediated transpositions. There are several strategies available to increase the frequency of remobilization events in the frog genome. Increasing the copy number of *SB *transposon substrates will likely significantly increase the frequency of novel re-integration events in the outcross progeny from hopper frogs. Transgenic frogs with multiple copies of the *pT2*βGFP transposon have been generated (7M and ♀622E) that each harbor more than seven copies of the *pT2*βGFP transposon [6]. The total number of transposon substrates in each hopper line can be further increased by incrossing the hopper lines with other *pT2*βGFP founders that contain multiple copies of the *SB *transposon. In addition to donor site transposon copy number, the transgenic transposase enzyme may also influence the remobilization activity in the frog. The transgenic *SB *enzyme frog described here was generated using the first generation *SB *transposase (*SB*10; [24]). In recent years, several hyperactive mutant forms of the *SB *enzyme have been developed, including *SB*11 that has approximately three-fold higher activity [48] and *SB*100X that has a 100-fold increase in enzymatic activity when compared to *SB*10 [49]. In addition to the choice of modified enzyme, different promoters with varying transcriptional activity could be used to drive expression of the *SB *transgene to enhance the rate of germline remobilization. This may not be as simple as finding the most powerful promoter and/or enhancer available, as *SB *is sensitive to overproduction inhibition, where increasing levels of enzyme impair the overall transposition efficiency [48].

### Why are different integration mechanisms observed with the co-injection and the double transgenic strategies?

There are several possible reasons for the different integration mechanisms used in the two *SB*-mediated methods, that is, injection-mediated and breeding-mediated transposition. First, the concentration of the substrate is vastly higher in the injection method where approximately 75 pg (around 10.8 × 10^6 ^copies) of the plasmid harboring the *SB *transposon substrate was co-injected with *SB *mRNA. By comparison, the *pT2*βGFP 8F founder used in the transgenic remobilization strategy contained three copies of the *SB *transposon. The *SB *transposase catalyzes transposition as a dimer of dimers (tetramer) bound to the indirect IR/DR elements that flank the transposon [32]. The massive excess of substrate present in the injection-mediated strategy may prohibit the correct assembly of transposase on the substrate and may result in non-canonical enzymatic activity. Also, the integrated transposon may also be a better substrate for *SB *activity due to DNA methylation and heterochromatinization [50,51]. Recent studies have shown that CpG methylation and supercoiling of *SB *transposon-harboring plasmids result in highly efficient transposition by the co-injection method in mammals when compared to non-methylated linear plasmid DNA donors [52]. Finally, the differences in the integration mechanisms observed with the two strategies may reflect differences in the availability of host factors for *SB *transposition in the developing gametes and the early-cleavage stage *Xenopus *embryos.

### Potential uses for *SB *remobilization in *X. tropicalis*

The demonstration that *SB *transposons stably integrated into the frog genome are effective substrates for remobilization is an important step in the development of large-scale insertional mutagenesis and enhancer- or gene-trap screens in the frog. The breeding-based remobilization strategy described here provides a simple and robust method for generating novel transgenic lines without the need for labor- and skill-intensive micro-injection methodologies. The frog provides several important advantages for transposon-based genetic screens. First, each outcross can generate several thousand progeny. The high fecundity of *X. tropicalis *indicates that, even if the remobilization frequency is low, multiple novel re-integration events can be identified in a single outcross.

A second advantage is that *Xenopus *have a long lifespan in captivity that may reach two decades or more, and the animals remain fertile for more than ten years. This has important implications for remobilization strategies, as double transgenic hopper frogs can be maintained and outcrossed at regular intervals for many years; male frogs can be outcrossed every two weeks and females every two months. Laboratories with limited animal holding space can, with a small cadre of hopper frogs, perform large-scale enhancer- or gene-trap screens, keeping only those tadpoles with interesting GFP expression profiles. The long lifespan of the hopper frogs may also have important implications if epigenetic silencing of the transgenic transposase locus is identified over a series of generations. Stably integrated transgenes are frequently subjected to epigenetic silencing over successive generations [12,53,54]. Silencing of the transposase locus would likely result in abolishment of the hopping activity. As each generation of frog lives for many years, having to regenerate new lines for hopping strategies is not likely to be a problem in this species.

The propensity of *SB *to catalyze local hopping events is a third advantage that can be exploited to generate insertional mutants of genes near the donor locus. In mice, approximately 75% of remobilized *SB *transposons re-integrate within 3 Mb of the donor locus [19]. Our data with remobilization of the 8F locus indicates that local hopping is also a feature of the *SB *transposition in *X. tropicalis*. Transgenic *SB *transposon frogs that have integrations in gene-dense regions of the genome can be used as donors for insertional mutagenesis strategies, as re-integration within a nearby gene may disrupt the normal activity of that locus. In the example presented here, the 8F transposon donor is located on scaffold 57, which maps to *X. tropicalis *chromosome 6 and is syntenic with human chromosome 7 (Figure 6a). It is in a gene dense region with approximately 50 genes in the 3 Mb flanking the transposon donor. The genome size of *X. tropicalis *is approximately one-half that of the human genome [3], while the gene content of the frog is similar to that of man. Thus, the overall gene density is relatively high in the frog. Different DNA 'cut and paste' transposon systems offer unique advantages for manipulating the vertebrate genome. For example, the local hopping activity of *SB *can be exploited to saturate the genomic sequences flanking the transposon donor locus with novel re-integration events. We have recently demonstrated that *Tol2 *transposons stably integrated into the frog genome are effective substrates for remobilization [7]. The local hopping activity of *Tol2 *is less pronounced than that of *SB*; approximately 20% of *Tol2 *re-integration events occur near the donor locus, compared to approximately 80% for *SB*. Using nested transposon substrates, with, for example, an *SB *transposon cloned within a *Tol2 *element, genome-wide remobilization screens could be performed using *Tol2 *to randomly distribute the dual substrate throughout the genome, with subsequent *SB *remobilization to locally saturate regions of interest with novel insertion events. In the study described here, we have used a simple ubiquitous promoter element to drive expression of the GFP reporter. Substrate transposons that harbor potentially more mutagenic elements, such as polyadenylation trap elements [55], can be used to efficiently disrupt the activity of the 'trapped' gene.

Finally, the frog is an excellent model for embryological and biochemical studies due to its small size, simple husbandry and the ease of manipulating embryos at all stages of development. Furthermore, as a tetrapod species, *X. tropicalis *shares a similar body plan with mammals, allowing analysis of developmental processes that are unique to higher vertebrates, such as limb and digit pattern formation. Combining these features with a simple and robust method for generating novel transgenic lines will provide valuable tools to apply to this highly tractable developmental model system.

## Conclusions

### *SB *transposons stably integrated into the *X. tropicalis *genome are substrates for remobilization

Co-injection of plasmid DNA harboring a *SB *transposon together with mRNA encoding the *SB *transposase results in efficient transgenesis of *X. tropicalis*. The integration events mediated by this co-injection approach are complex and frequently contain low-order concatemers of the transposon. In this study, we demonstrate that *SB *transposons stably integrated in the frog genome are effective substrates for remobilization. Transgenic frogs that express *SB*10 transposase in the germline were bred with *SB *transposon frogs and the double transgenic progeny were outcrossed to wild-type animals. Remobilization events were readily identified by increased GFP expression in the offspring where the parental transposon concatemer had been modified by the *SB*10 enzyme. Integration site analysis of the GFP-bright progeny indicated that the transposon re-integration events had occurred via a canonical cut and paste mechanism. The rate of remobilization observed in the frog was similar to that observed in other species when a low copy number concatemer was used as the transposon donor.

### *SB *transposon remobilization as a tool for genetic manipulation of *X. tropicalis*

The diploid frog *X. tropicalis *offers several advantages for large-scale forward genetic screens in a tetrapod model, including vast numbers of progeny per spawn, long lifespan and availability of genomic resources including the genome sequence and genetic map. Here, we have demonstrated that we can exploit the cut and paste activity of *SB *transposase to generate novel transposon transgenics by simply breeding the double-transgenic hopper frogs. Novel transposon lines are readily identified by changes in GFP reporter expression in the remobilized progeny compared to the parental GFP pattern. The local hopping activity of *SB *can be exploited to saturate genomic regions that flank the transposon donor sites. The ability to generate thousands of progeny in each outcross, combined with the ease of identifying novel insertion events, will allow large-scale *SB*-mediated screens to be performed in *X. tropicalis*.

## Methods

### Plasmids and generation of transgenic lines

The generation of the *pT2*βGFP construct and the transgenic *pT2*βGFP *X. tropicalis *line 8F have been described previously [6]. The pCAGGS-*SB*10 construct was a gift from Dr David Largaespada [27]. A 3,613 bp *Hin*cII/*Bam*HI fragment containing the promoter/enhancer and the *SB*10 transposase from pCAGGS-*SB*10 was cloned in pBluescript SK+ (pBS-SK+) to generate pBS-CAGGS-*SB*10. The 2.2 kb *X. laevis *γ1 crystallin promoter driving dsRed construct was a gift from Dr Robert Grainger (2.2 γ1 crystallin-RFP; [28]). An approximately 3.5 kb γ1-crystallin promoter-RFP fragment was PCR amplified from 2.2 γ1 crystallin-RFP using primers DSR1 5'-GTAAGCGGCAGGGTCGGA-3' and DSR2 5'-GCCTCGAGCGATTTCGGCCTATTGGT-3', cloned into pGEM-Teasy (Promega, Madison, WI, USA), and fully sequenced, to yield pGEM-γcRFP. An approximately 3.5 kb *Sac*I restriction fragment from pGEM-γcRFP encoding the γ1 crystallin-RFP reporter was cloned into the unique *SacI *restriction site of pBS-CAGGS-*SB*10. A single clone was selected with the two mini-genes oriented tail-to-tail in the pBS-SK plasmid (pCAGGS-*SB*10;γcRFP; Figure 1a). The pCAGGS-*SB*10;γcRFP construct was linearized with *Sca*I and injected *in vitro *into *X. tropicalis *fertilized embryos at the one-cell stage (500 pg of linear plasmid DNA in 3 nL of water) as described previously [6,8]. Tadpoles were scored for expression of RFP in the lens after stage 40 [30]. RFP-positive tadpoles (27 positive from 570 injected, 4.7%) were selected and raised to adulthood and outcrossed to determine germline transmission of the transgene. Male frog CAGGS-*SB*10;γcRFP 2M produced progeny that expressed RFP robustly in the lens and was selected for further analysis.

### Husbandry and micro-injection of *X. tropicalis*

*X. tropicalis *tadpoles were maintained at 28°C in static tanks and were staged according to Nieuwkoop and Faber [30]. Adult animals were housed in a recirculating aquarium at 26°C. Transgenic adult frogs were identified by implanting a radio-frequency identification microchip (microSensys GmbH, Erfurt, Germany) beneath the skin of the dorsal surface of each animal [56]. The unique 16-digit alphanumeric sequence encoded on each chip provides a convenient method for identifying individual animals throughout their lifespan. Female *X. tropicalis *animals were pre-primed with a 1:5 dilution of human chorionic gonadotropin (hCG) overnight, and primed the day of injection with 200 U of hCG. Fertilized eggs were obtained by natural matings. Injected eggs were allowed to heal at 28°C and transferred to tanks for growth at 28°C [56]. This project was approved by St. Jude Children's Research Hospital's Institutional Animal Care and Use Committee.

### RT-PCR analysis of *SB *expression

Total cellular RNA was isolated using the RNAeasy kit (Clontech, Mountain View, CA, USA) from individual RFP-positive and RFP-negative stage 40 embryos generated by outcross of CAGGS-*SB*10;γcRFP 2M. First strand cDNA was synthesized and used as template for ^32^P-labelled PCR reactions as described previously [57]. Primers for *SB*10 amplification (SB3 5'-GCCGCTCAGCAAGGAAGA-3' and SB4 5'-GAAGACCCATTTGCGACCAAG-3') annealed at 56°C and produced a 383 bp fragment. Primers to *Xenopus *α-actin were used as a control for RNA recovery.

### Western blot analysis of *SB*10 protein in tissues harvested from transgenic frogs

Whole embryo or adult tissue samples were snap frozen in a dry ice and ethanol bath and stored at -80°C. 100 μl of RIPA buffer (150 mM sodium chloride, 50 mM tris(hydroxymethyl)aminomethane with hydrogen chloride pH 7.5, 1% (v/v) nonyl phenoxypolyethoxylethanol (IPEGAL), 0.5% (w/v) sodium deoxycholate, 0.1% (w/v) sodium dodecyl sulfate, 1X Complete Protease Inhibitor Cocktail (Roche, Indianapolis, IN, USA)) was added to the frozen samples, mixed by vortex, extracted with Freon (200 μL of 1,1,2-trichlorotrifluroethane (Sigma-Aldrich, St. Louis, MO, USA)) and centrifuged at 16,100 × *g *for 15 minutes at 4°C. The upper phase was transferred to a fresh tube and the protein concentration was measured using the Bradford assay (Bio-Rad, Hercules, CA, USA). Aliquots of each sample were diluted with an equal volume of 2× Laemmli Sample Buffer containing β-mercaptoethanol (Bio-Rad) and denatured by heating at 100°C for 5 minutes. Proteins were separated by electrophoresis on 4% to 15% (w/v) Criterion precast polyacrylamide gels (Bio-Rad); pre-stained SDS-PAGE standards (Bio-Rad) were used as molecular weight markers. Proteins were transferred to Hybond-P polyvinylidene difluoride (PDVF; GE Healthcare Life Sciences, Piscataway, NJ, USA) membranes at 50 V for 1.5 hours at 4°C. Protein transfer was verified by staining the membrane with Ponceau S. Monoclonal anti-*SB *transposase antibody (MAB2798; R&D Systems, Minneapolis, MN, USA) was resuspended at a final concentration of 500 μg/mL and diluted 1:500 to probe the membranes blocked with Superblock (Pierce, Rockford, IL, USA) containing 0.05% (v/v) Tween 20. A secondary goat anti-mouse horseradish peroxidase-conjugated antibody was diluted to 1:10,000 and developed using the Chemiluminescent Detection System (Pierce). Membranes were stripped with Restore Western Blot Stripping Solution (Pierce) and re-probed with a mouse monoclonal antibody specific for *Xenopus *β-actin (ab8224; Abcam, Cambridge, MA, USA) as a control for protein recovery.

### Fluorescent protein expression analysis

A Leica FLIII fluorescent dissecting microscope was used to analyze GFP and RFP expression. Digital images were captured using a Nikon Ri1 color digital camera and the Nikon Elements Basic Research software package (Nikon, Melville, NY, USA). Tadpoles were immobilized for photography by brief anesthesia in 0.015% (w/v) tricaine methanesulphonate.

### Southern blot hybridization

Genomic DNA was harvested from individual tadpoles by overnight proteinase K digestion at 56°C and phenol/chloroform/isoamyl alcohol extraction using standard protocols [8]. Genomic DNA (3 μg to 5 μg) was digested with *Bgl*II, separated on a 0.7% (w/v) agarose gel and transferred to Hybond N+ hybridization transfer membranes (GE Healthcare Life Sciences, Piscataway, NJ, USA). The hybridization membranes were probed with a ^32^P-radiolabeled fragment of the GFP open reading frame (approximately 700 bp) and exposed onto a GE Healthcare Life Sciences phosphorimager screen for detection.

### Genomic PCR and transposon integration site analysis

Integration site analysis was performed EPTS LM-PCR to the right arm (IR/DR) of the *SB *transposon as described previously [6,8]. The integration sites were verified using genomic PCR strategies with primers that bind to scaffold sequences beyond the EPTS LM-PCR products. PCR primers were also designed to amplify the predicted sequences that flank the left IR/DR of the *SB *transposon. All genomic PCR products were cloned into either pGEM-T Easy (Promega) or TOPO-TA (Invitrogen, Carlsbad, CA, USA) and sequenced. Novel sequences were queried against the Joint Genome Institute *X. tropicalis *genome (version 4.1; http://genome.jgi-psf.org/Xentr4/Xentr4.home.html) and scaffolds were assigned to chromosomes and linkage groups based on the genetic map developed at the University of Houston [44].

## List of abbreviations

bp: base pair; CAGGS: chicken β-actin promoter coupled with a cytomegalovirus enhancer; EPTS LM-PCR: extension primer tag selection linker-mediated polymerase chain reaction; FISH: fluorescence *in *situ hybridization; GFP: green fluorescent protein; hCG, human chorionic gonadotropin; IR/DR: indirect repeat/direct repeats; PCR: polymerase chain reaction; RFP: red fluorescent protein; RT: reverse transcriptase; SB: *Sleeping Beauty*; UTR: untranslated region.

## Competing interests

The authors declare that they have no competing interests.

## Authors' contributions

DAY carried out embryo injections, scored tadpoles, performed molecular analysis of transposon integration sites and helped prepare the manuscript. CMK performed molecular analyses of transposon integration sites, scored tadpoles and helped prepare the manuscript. EK performed embryo injections, scored progeny, assisted with molecular analyses and helped with general husbandry. HZ performed embryo injections and helped score progeny. MRJH generated the *SB*10 transposase transgenic line. AKS and DEW provided mapping data to assign sequence scaffolds to the *X. tropicalis *linkage groups and/or chromosomes. PEM conceived the study, directed the project and wrote the manuscript. All authors read and approved the final manuscript.
